# Gene-level analysis of core carbohydrate metabolism across the *Enterobacteriaceae* pan-genome

**DOI:** 10.1038/s42003-025-08640-5

**Published:** 2025-08-18

**Authors:** Nicolas Näpflin, Christopher Schubert, Lukas Malfertheiner, Wolf-Dietrich Hardt, Christian von Mering

**Affiliations:** 1 Department of Molecular Life Sciences and Swiss Institute of Bioinformatics, University of Zurich, Zurich, Switzerland; 2 Institute of Microbiology, D-BIOL, ETH Zurich, Zurich, Switzerland

**Keywords:** Microbial genetics, Genome informatics

## Abstract

*Enterobacteriaceae* is a diverse bacterial family that commonly colonizes the gastrointestinal tracts of humans and animals, influences host health, and also includes members adapted to colonize the phyllosphere as well as insect hosts. We lack systematic knowledge regarding the core metabolic strategy shared among *Enterobacteriaceae*. To address this gap, we have analyzed the pan-genome of nearly 20,000 genomes, including *Citrobacter*, *Escherichia*, *Klebsiella*, and *Salmonella*. We found that genes necessary for monosaccharide-fuelled mixed acid fermentation and (micro-)aerobic respiration are part of the *Enterobacteriaceae* core genome, whereas most genes involved in anaerobic respiration and carbohydrate utilization are associated to the accessory genome. Most *Enterobacteriaceae* possess genes enabling the utilization of D-glucose, its epimers, D-glucose-containing disaccharides, and chemically modified derivatives of D-glucose - highlighting the evolutionary adaptation of this family to efficiently exploit this simple sugar. Understanding *Enterobacteriaceae*’s core metabolic strategy helps clarify the distinction of niche-defining nutrient sources, which can be genus-, species- or strain-specific. This study highlights the core metabolic strategy of *Enterobacteriaceae*, supporting the development of targeted interventions in microbiome research and infectious disease control.

## Abstract (web summary)

Pan-genome analysis reveals that *Enterobacteriaceae* share a core metabolic strategy based on monosaccharide-fueled mixed acid fermentation and aerobic respiration, enabling future identification of niche-defining traits in this diverse bacterial family.

## Introduction

The *Enterobacteriaceae* family consists of diverse Gram-negative bacteria, including both harmful pathogens and commensal members. This family plays a critical role in public health and veterinary medicine, as it is responsible for a large number of foodborne infections worldwide^1–4^. A defining trait of *Enterobacteriaceae* is their facultative anaerobic lifestyle, allowing them to thrive with or without oxygen. They play an important role in early neonatal life by consuming oxygen in the gut, thereby creating beneficial conditions for obligate anaerobe colonization^5,6^. On the other hand, *Enterobacteriaceae* can also play a prominent role in disease, e.g., in inflammatory bowel disease^7,8^ or in diarrheal infections^4^. Extensive research has focused on the virulence factors of pathogenic *Enterobacteriaceae* within genera such as *Escherichia*, *Salmonella*, *Citrobacter*, and *Shigella*, which can cause disease in a wide range of mammals^9–12^. In addition to the gastrointestinal tract, genera such as *Enterobacter* and *Serratia* have also been identified in the phyllosphere^13^. Moreover, *Serratia symbiotica* is a well-established symbiont in insects^14^. Members of the *Enterobacteriaceae* family are adapted to a broad range of environmental reservoirs (see for review Janda and Abbott 2021). To sharpen the focus of this study, we conducted a gene-level analysis of core carbohydrate metabolism in *Enterobacteriaceae*, with particular emphasis on interpreting the data in the context of the gut as a primary habitat for many genera within this family.

Growth in the animal gut lumen is important for commensal and pathogenic *Enterobacteriaceae* alike. However, it is still not fully understood how *Enterobacteriaceae* strains can establish themselves in a gut that has already been colonized by another related strain. Rolf Freter’s concept of nutrient niches posits that two related bacterial strains can only co-exist if each occupies a preferred niche based on its superior use of a specific nutrient in a well-mixed environment^15,16^. This theory extends to spatial structuring within the gut, known as the Restaurant Hypothesis^17–20^. Nutrient competition among microbiota is a key factor in colonization resistance, where the gut microbiota helps protect against invading bacteria^21^. The central principle is metabolic resource overlap, where microbes with overlapping metabolic needs compete for the same nutrients, effectively blocking access for others^22^. This nutrient competition, which is shaped by the diversity of the microbial community, is particularly effective when the microbiota comprises members closely related to the invading pathogen^22,23^. However, our understanding of how widely metabolic characteristics are shared among the *Enterobacteriaceae* family remains limited.

Pan-genome analysis assesses the entire genomic repertoire of a bacterial clade - a widely used approach to explore genetic diversity^24,25^, evolutionary relationships^26,27^, metabolic capabilities^28–30^, and the distribution of core and accessory genes^31,32^. The core genome consists of genes present in most genomes, while the accessory genome includes lineage-specific and unique genes (singletons), which may be acquired through horizontal gene transfer. The accessory genome is a key source of functional and genetic diversity^33^. Many bacterial species exhibit an open pan-genome, which tends to expand as more genomes are analyzed^34,35^. This underscores the need for comprehensive datasets to capture the genomic diversity in the most comprehensive fashion. Advancements in high-throughput sequencing have led to an increase in available genomes^36,37^.

Previous studies on bacterial pan-genomes have predominantly highlighted differences rather than commonalities, often concentrating on species-specific distinctions. We deliberately included *Enterobacteriaceae* associated with the gut, phyllosphere, insects, and other environmental reservoirs in our pan-genome analysis to uncover the core metabolic strategies shared across this ecologically diverse bacterial family. Our aim was to identify the context-independent metabolic strategy employed by *Enterobacteriaceae*, regardless of the reservoir they inhabit. We utilized a bioinformatics approach to categorize core and accessory genes across an extensive dataset of *Enterobacteriaceae* genomes. Our analysis covered 16 *Enterobacteriaceae* genera, such as *Salmonella*, *Escherichia*, *Klebsiella*, *Yersinia*, *Enterobacter*, and *Shigella*. Future studies can use this information to determine whether the genetic traits that enable *Enterobacteriaceae* isolates to inhabit different environmental reservoirs represent a conserved strategy or are genus- or species-specific.

## Results

### Pan-genome analysis and phylogenetic context of *Enterobacteriaceae*

**Fig. 1:**
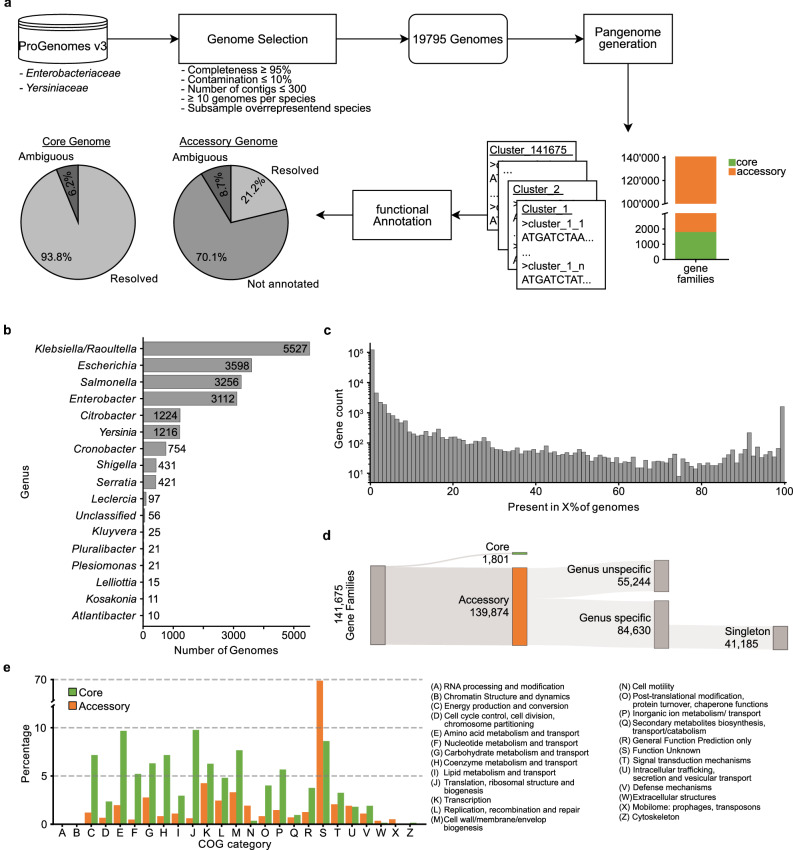
Overview of the bioinformatics workflow. **a** The *Enterobacteriaceae* pan-genome currently includes 141’675 gene families. Gene families are grouped based on their annotation status: Resolved (≥90% of the sequences contain the same annotation), ambiguous (<90% of the sequences contain the same annotation and <10% unknown), and not annotated (≥10% of the sequences are not annotated). **b** A total of 19,795 *Enterobacteriaceae* genomes across 16 genera were analyzed. **c** Gene ubiquity distribution (expressed as fraction of genomes containing a gene) following a U-shaped curve. **d** Distribution of gene families into core and accessory genomes. The accessory genome is further split into genus-specific and unspecific families. Almost 50% of genus-specific gene families are unique to a single genome (singleton). **e** The functional categories of the accessory genome are dominated by gene families with unknown functions (68.2%). The most frequent functions in the core gene families are Translation (9.8%), Amino acid metabolism and transport (9.7%) and cell wall/membrane/envelope biogenesis (7.7%).

We analyzed the pan-genome of *Enterobacteriaceae* across 16 genera and 80 species, encompassing nearly 20,000 high-quality genomes (≥95% completeness, ≤5% contamination) sourced from progenomes3^38^ (Supplementary Data 1, Fig. 1a). Genomes from overrepresented species (e.g., *Escherichia coli*, *Klebsiella pneumoniae*, *Salmonella enterica*) were randomly subsampled, and species with fewer than 10 genomes were excluded (Fig. 1b, Supplementary Data 2). Orthologous gene families were identified across a range of amino acid identity thresholds (50–95%, in increments of 5%, see Methods), resulting in 141,675 gene families clustered from almost ~95 million sequences. Gene ubiquity followed a universal U-shaped distribution^39^, with only 1.2% of gene families (1801) forming the core genome (present in ≥95% of genomes). Nearly 60% of gene families were genus-specific, from which 48% were singletons, reflecting high intra-family diversity (Fig. 1c, d). Functional annotation was performed using the UniProtKB/Swiss-Prot database^40^ (Fig. 1a). The more ubiquitous gene families tend to be well annotated, while rare gene families (present in less than 5% of genomes) make up over 90% of the pan-genome and remain largely unannotated. Finally, we annotated all genes with Clusters of Orthologous Groups (COG) functional categories using eggNOG-mapper^41^ to obtain broad functional categories at the gene family level. Core genes were enriched in translation, amino acid metabolism, and membrane biogenesis, whereas accessory genes were largely unannotated and fell predominantly into the function unknown COG category (Fig. 1e). To provide phylogenetic context, we constructed a core genome tree using 103 concatenated proteins (Supplementary Fig. 1, Supplementary Data 3), confirming established relationships among core *Enterobacteriaceae* genera and the close phylogenetic proximity of *Escherichia*, *Citrobacter*, and *Salmonella*^42–45^. Our tree also supports the polyphyletic nature of *Shigella*, reinforcing its classification within *Escherichia*^46–48^, and the genera *Plesiomonas*, *Yersinia*, and *Serratia* branch off relatively early in relation to *Escherichia*^1,49^. In conclusion, these findings are consistent with previous 16S rRNA and multilocus phylogenetic studies^1,45,49,50^.

### *Enterobacteriaceae* are adapted to abundant monosaccharides in nature

**Fig. 2:**
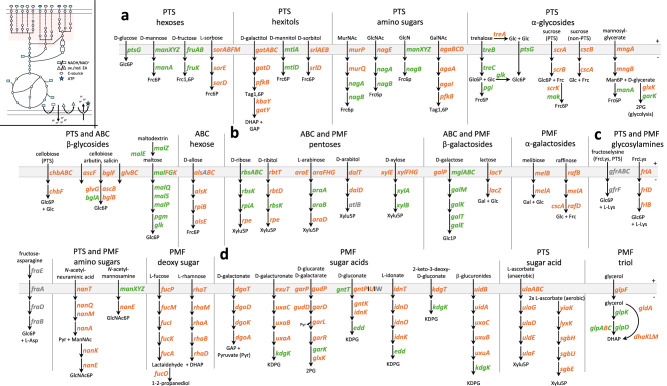
Core and accessory gene distribution of carbohydrate degradation pathways of *Enterobacteriaceae.* A gene family is deemed to be ‘core’ when it is present in at least 95% of all analyzed *Enterobacteriaceae* genomes (green). A prevalence of less than 95% is defined as accessory (orange). Genes that were not found in the analysis but have experimental evidence are shown in gray. The top left subpanel indicates energy metabolism, with the respective part of interest marked in red. **a** Depiction of carbohydrates that are almost exclusively transported by the bacterial phosphotransferase system (PTS). Panels **b**, **c** show a mixture of pentoses, hexoses, disaccharides, glycosylamines, and amino sugars that are transported either through primary transport by an ATP-binding cassette (ABC) or via secondary transport through a proton-motive force (PMF)-driven system. **d** Depiction of hexuronate utilization driven by PMF transporters, and the triol glycerol. The different substrate groups are indicated above the metabolic pathways. The abbreviations of carbohydrates are defined in Supplementary Data. 3.

Our core genome phylogeny confirms the expected clustering of *Enterobacteriaceae* into distinct genera (Supplementary Fig. S1), providing a taxonomically broad dataset for investigating metabolic strategies. Since our aim is to investigate context-independent metabolic strategies of *Enterobacteriaceae* across diverse environmental reservoirs, we focused specifically on carbohydrate utilization and energy metabolism. In the following sections, we illustrate how these metabolic strategies manifest during gut colonization - a key habitat for selected genera of *Enterobacteriaceae*. Nutrient exploitation is one of the key functions of colonization resistance provided by the microbiome^19,51,52^. Recent publications have highlighted the importance of *Enterobacteriaceae*-*Enterobacteriaceae* competition in effectively limiting or even preventing colonization^22,53–58^. This competition is often governed by the differential metabolic utilization of specific carbohydrates. We performed a gene-level analysis of our pan-genome dataset to examine energy metabolism, beginning with carbohydrate utilization (Fig. 2).

The main carbohydrate transport systems are the phosphotransferase system (PTS), ATP-binding cassette (ABC), and proton motive force (PMF)-driven transport systems (Fig. 2). Carbohydrates transported by the PTS, such as D-glucose, are preferred by *Enterobacteriaceae*^59^. Genes required for degrading PTS sugars, such as D-glucose, D-mannose, D-fructose, *N*-acetylglucosamine, and D-glucosamine, are found within the core genome (Fig. 2a, green). The degradation of hexitols mainly belongs to the accessory genome (Fig. 2a, orange), except for D-mannitol (MtlA, MtlD) (Fig. 2a). Hexitols and other sugar alcohols present a metabolic challenge under anaerobic conditions of the animal gut, when no external electron acceptor is available, as they generate a reducing equivalent before entering glycolysis. The oxidation of a hydroxyl group (–OH) during sugar cyclization produces an additional reducing equivalent that cannot be disposed of through exclusively mixed acid fermentation. The only disaccharides associated with the core genome are trehalose and maltose, both composed of D-glucose molecules but differing in their glycosidic linkages: trehalose has a 1,1-linkage, while maltose features a 1,4-linkage (Fig. 2a). D-ribose is the only pentose whose transport and degradation belong to the core genome. Unlike D-ribose, L-arabinose and D-xylose can be transported by two distinct systems: a low-affinity PMF-driven transporter and a high-affinity ABC transporter, both of which are associated with the accessory genome (Fig. 2b)^60^. The majority of cytosolic enzymes for degrading L-arabinose and D-xylose are found within the core genome. Thus, L-arabinose and D-xylose utilization is common among *Enterobacteriaceae*, though import occurs via diverse transporters (Fig. 2b). In particular, for *Salmonella*, we found that the presence of the low-affinity L-arabinose transporter and absence of the high-affinity transporter correlates with the presence of an arabinofuranosidase capable of releasing L-arabinose from arabinan, its polymeric form (refer to Fig. 5e, f). Notably, the D-glucose epimer D-galactose is associated with the core genome (Fig. 2b). Glycosylamines, which consist of a reducing sugar and an amino moiety, are produced during food processing through the Maillard reaction under heat, forming compounds such as fructose-asparagine and fructose-lysine (Fig. 2c). Amino sugars are sugar molecules in which a hydroxyl group is replaced with an amino group, such as D-glucosamine, D-mannosamine, and neuraminic acid. These sugars are also commonly found in their acetylated forms, such as N-acetylglucosamine. Only the pathway for N-acetylglucosamine (transported via ManXYZ) and D-glucosamine degradation are fully included in the core genome (Fig. 2a, c). Sugar acids, which are hexoses with a carboxyl group (–COOH) at the end of the chain, generally have their degradation genes included in the accessory genome (Fig. 2d). Reactive nitrogen species in the gut can oxidize hexoses during post-antibiotic stress, converting D-glucose to D-glucarate, for example^61^. Additionally, sugars like D-galacturonic acid, D-glucuronic acid, and L-iduronic acid naturally occur in various complex polysaccharides^62^.

*Enterobacteriaceae* analyzed in this study share a small number of carbohydrate uptake and degradation systems that are components of the core genome, which are focused on D-glucose, D-glucose epimers, D-glucose-containing disaccharides, and modified D-glucose molecules. This partly mirrors mammalian cells, which have evolved to preferentially utilize glucose^63^.

### The three glycolytic pathways are associated with the core genome

Most carbohydrate utilization pathways in *Enterobacteriaceae* are linked to the accessory genome (Fig. 2). However, the majority of pathways for phosphotransferase system (PTS)-transported carbohydrates - such as D-glucose, D-mannose, D-fructose, D-mannitol, N-acetylglucosamine, and trehalose - that route these carbohydrates into glycolysis are found in the core genome (Fig. 2a).

**Fig. 3:**
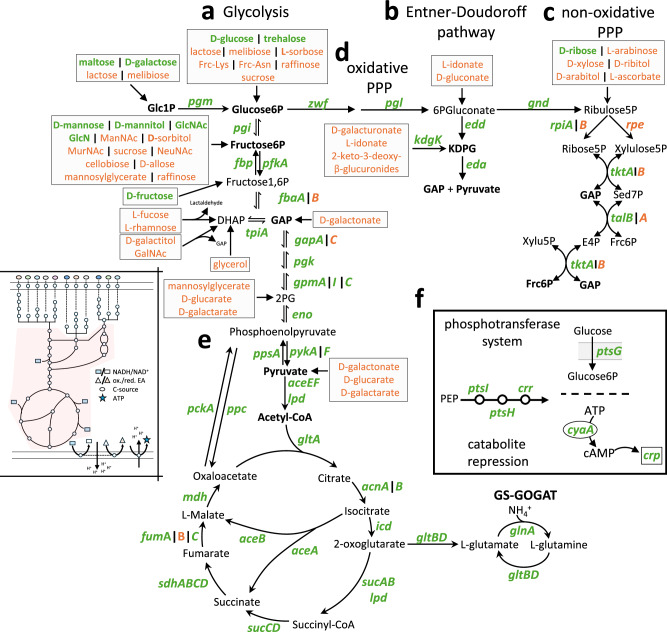
Core and accessory gene distribution in the glycolytic pathways and TCA cycle of *Enterobacteriaceae.* Core genes are defined as present in at least 95% of all analyzed *Enterobacteriaceae* genomes (green). A prevalence of less than 95% is defined as accessory (orange). The left subpanel indicates energy metabolism, with the respective part of interest marked in red. The three glycolytic pathways - **a** glycolysis, **b** Entner–Doudoroff pathway, and **c** non-oxidative pentose phosphate pathway (PPP) - are shown, including **d** the oxidative PPP, which connects glycolysis to the non-oxidative PPP. The entry points for the various carbohydrate sources from Fig. 2 are indicated by gray boxes. The color-coding shows whether the utilization pathway is part of the core (green) or accessory (orange) genome of *Enterobacteriaceae*. **e** Tricarboxylic acid (TCA) cycle, including the carbon-saving glyoxylate shunt and the glutamine synthetase (GS) - glutamate 2-oxoglutarate aminotransferase (GOGAT) pathway. **f** Depiction of the phosphotransferase system (PTS), which uses phosphoenolpyruvate (PEP) as a phosphoryl group donor to phosphorylate incoming PTS-transported sugars in a process known as group translocation. Furthermore, the EIIA^Glc^ (*crr*) stimulation of adenylyl cyclase (*cyaA*), which largely controls catabolite repression via cAMP-CRP signaling, is indicated. The abbreviations of carbohydrates are defined in Supplementary Data 3.

The three glycolytic pathways - namely, glycolysis, the Entner–Doudoroff pathway, and the non-oxidative and oxidative pentose phosphate pathways (PPP) - as well as the tricarboxylic acid (TCA) cycle, are all part of the core genome (Fig. 3a–e). Glycolysis, a universal pathway present in both prokaryotes and eukaryotes, metabolizes glucose into two molecules of pyruvate while generating NADH and ATP (Fig. 3a)^64^. In contrast, the Entner-Doudoroff pathway in *Enterobacteriaceae* converts hexuronates, while the pentose phosphate pathway processes pentoses into glycolytic intermediates, enabling carbon flux into the TCA cycle (Fig. 3b, c). The oxidative pentose phosphate pathway links glycolysis and the Entner-Doudoroff pathway to the non-oxidative pentose phosphate pathway. This pathway provides NADPH for biosynthesis and contributes to the non-oxidative pentose phosphate pathway for biosynthetic precursors (Fig. 3d)^65^. The TCA cycle provides energy and reducing equivalents, and biosynthetic precursors. The glyoxylate shunt, involving AceA and AceB, allows bypassing of the NADH/NADPH-producing steps (Icd and Lpd–SucAB) as well as the decarboxylation steps of the TCA cycle^66^. This is particularly important during growth on acetate, where the *acs* pathway channels carbon into central metabolism without carbon loss as CO_2_^67^. The regulatory pathway that governs carbohydrate utilization is the PTS, which primarily affects catabolite repression and phosphorylates incoming PTS-transported carbohydrates via a process known as group translocation^59^ (Fig. 3f). In addition, the PTS impacts cyclic AMP-dependent catabolite repression, aligning bacterial proteome allocation with metabolic needs^68^. The PTS acts as the central regulatory circuit, allowing *Enterobacteriaceae* to optimize growth by adapting to varying carbon sources through a complex hierarchical logic, which has been studied in great detail for *E. coli* K12^69^.

Interestingly, some isozymes - enzymes that catalyze the same reaction - are linked to the accessory genome, while the primary enzyme is part of the core genome. For example, in *E. coli*, TktA (core) is the main enzyme in the non-oxidative pentose phosphate pathway, while TktB (accessory), an isozyme, has only minor activity^70^ (Fig. 3c). Conversely, *pykF* and *pykA*, encoding pyruvate kinase I and II, respectively, are both associated with the core genome, likely due to their differing physical and chemical properties and kinetic behavior. For example, PykF is allosterically activated by fructose 1,6-bisphosphate, while PykA is activated by AMP (Fig. 3a)^71^. Similarly, fumarases A, B, and C exhibit distinct properties: FumA is a dimeric iron-sulfur cluster-containing hydrolase expressed aerobically and anaerobically, FumB is expressed anaerobically, and FumC is an iron-independent fumarase induced during oxidative stress^72,73^. *fumA* and *fumC* are part of the core genome, while *fumB* is not (Fig. 3e). The gene *fumB* is often colocalized with *dcuB*^74^, which is classified as part of the core genome. Therefore, the association of *fumB* with the accessory genome likely reflects a limitation of our annotation pipeline rather than a biologically meaningful observation, potentially due to ambiguous resolution among closely related fumarase genes such as *fumD* and *fumE*, which are also associated with the accessory genome.

In conclusion, the three glycolytic pathways that provide NADH, NADPH, ATP, and important precursors for biosynthesis are part of the *Enterobacteriaceae* core genome. Isozymes without distinct enzymatic properties are associated with the accessory genome. In contrast to carbohydrate utilization, central carbon metabolism is highly conserved in *Enterobacteriaceae*.

### Respiration of inorganic electron acceptors is associated with the accessory genome

**Fig. 4:**
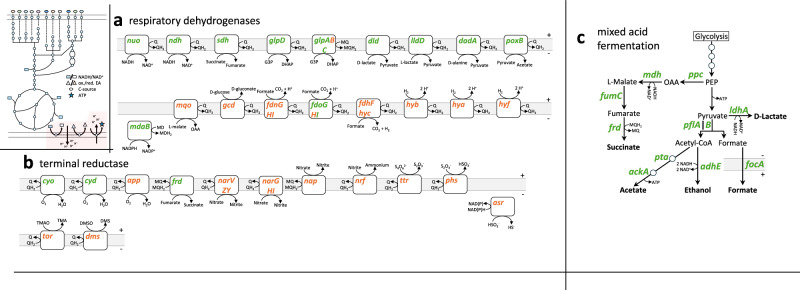
Core and accessory gene distribution of respiratory dehydrogenases, terminal reductases, and mixed acid fermentation in *Enterobacteriaceae.* A core gene is present in at least 95% of all analyzed *Enterobacteriaceae* genomes (green). A prevalence of less than 95% is defined as accessory (orange). The top left subpanel indicates energy metabolism, with the respective part of interest marked in red. **a** Depiction of respiratory dehydrogenases that oxidize an electron donor (NADH, succinate, etc.) and transfer the electrons into the quinone pool, thereby reducing quinone (Q and MQ, oxidized state) to quinol (QH_2_ and MQH_2_, reduced state). Q can stand for ubiquinone or menaquinone (MQ). Menadione is abbreviated MD. **b** Terminal reductases oxidize quinols and transfer the electrons to an electron acceptor, thereby balancing the redox reaction. The reduced electron acceptor is typically an end product and is secreted. **c** The alternative pathway for redox balancing and ATP formation in the absence of an inorganic electron acceptor is mixed acid fermentation, which exclusively relies on carbon-based electron donors and acceptors for energy and redox balance. The abbreviations of sugars and the complete list of individual genes in the respective complexes (e.g., *hyb*) are defined in Supplementary Data 3 and 4.

The three glycolytic pathways break down carbohydrates for anaplerosis, supplying important precursors for biosynthetic pathways and generating ATP and NADH for energy metabolism. Energy is conserved in the form of ATP, either by substrate-level phosphorylation or through oxidative phosphorylation. During oxidative phosphorylation, electrons are transferred through a series of redox reactions from a carbon-based electron donor, such as D-glucose, to an organic or inorganic electron acceptor, such as oxygen. Electrons are temporarily stored in the reduced forms of NAD^+^ and the quinones (reduced state: NADH and quinols). Respiratory dehydrogenases, such as *nuo* and *ndh*, oxidize NADH and reduce the quinone pool. In addition to NADH (*nuo* and *ndh*) and succinate (*sdh*) dehydrogenases, which are associated with the core genome, other respiratory dehydrogenases include those for glycerol 3-phosphate (*glpD*), D-lactate (*dld*), L-lactate (*lldD*), D-alanine (*dadA*), pyruvate (*poxB*), and NADPH (*mdaB*) (Fig. 4a). Quinols transfer electrons to a terminal reductase that reduces an electron acceptor (Fig. 4b). The aerobic (*cyo*) and the microaerobic (*cyd*) oxygen oxidoreductases, as well as the fumarate reductase (*frd*) are the only terminal reductases included in the core genome. The remaining terminal reductases, which utilize inorganic electron acceptors such as nitrate or tetrathionate (S_4_O_6_^2^^−^), are part of the accessory genome of *Enterobacteriaceae* (Fig. 4b). In brief, this encompasses the electron transport chain, coupled with the translocation of protons to establish a proton motive force, which is utilized, for instance, in ATP production via ATP synthase (oxidative phosphorylation) or in secondary transport^75^.

Alternatively, *Enterobacteriaceae* can maintain redox balance and produce ATP during mixed acid fermentation (Fig. 4c). This process, a defining feature of facultative anaerobic *Enterobacteriaceae*, produces a range of acidic end products: succinate, acetate, formate, D-lactate, and the non-acidic ethanol. Acetate fermentation conserves energy in the form of ATP, while succinate, ethanol, and D-lactate fermentation regenerate NAD^+^ and quinones. The acidic and non-acidic end products are exported from the cell, while formate can be used as an electron donor via a respiratory dehydrogenase (*fdn*, *fdo*, and *fdh-hyc*) (Fig. 4a, c). The pivotal enzyme in this process is pyruvate formate lyase (PflB), which catalyzes the non-oxidative cleavage of pyruvate into acetyl-CoA and formate. This step is more carbon- and redox-efficient compared to the aerobic counterpart, pyruvate dehydrogenase (*aceEF lpd*), which produces carbon dioxide and NADH. All genes involved in the key steps of mixed acid fermentation belong to the core genome (Fig. 4c).

In conclusion, *Enterobacteriaceae*, which are predominantly facultative anaerobic bacteria, possess genes for both aerobic respiration and mixed acid fermentation within their core genome (Fig. 4). The distribution of respiratory dehydrogenases and terminal reductases between the core and accessory genomes mirror the distribution of carbohydrate utilization pathways (Figs. 2 and 4). Both types of pathways serve as key interfaces within an ever-changing environment.

### How are the carbohydrate utilization pathways distributed between different strains?

**Fig. 5:**
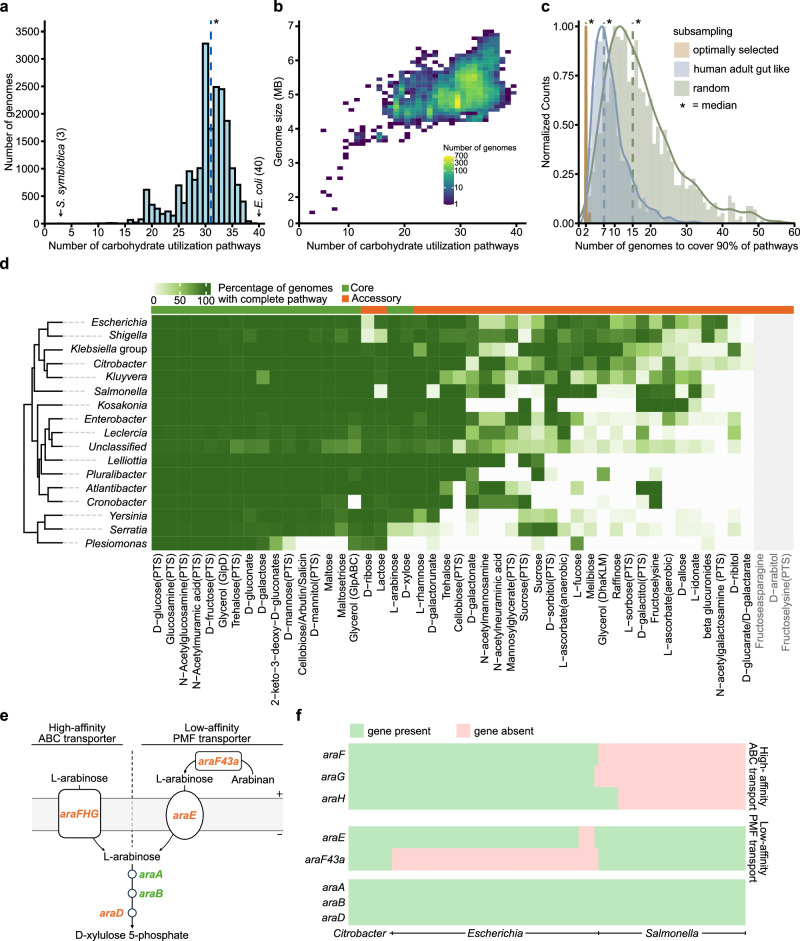
Distribution of carbohydrate utilization pathways in *Enterobacteriaceae.* **a** *Enterobacteriaceae* genomes contain, on average, 31 carbohydrate utilization pathways (blue dashed line) out of the 49 investigated. **b** The number of carbohydrate utilization pathways is weakly correlated with genome size. Spearman’s correlation: 0.49, *P*-value < 2.2E−16. **c** Subsampling all investigated genomes while assuming equal probability for each genus, 15 randomly selected *Enterobacteriaceae* strains are, on average, sufficient to cover 90% of carbohydrate utilization pathways (random, green dashed line). Selecting genomes based on the distribution of *Enterobacteriaceae* in a healthy human adult gut, 7 genomes are typically required (human adult gut-like, blue dashed line). Constructing a minimal set of genomes to cover 90% of the pathways requires just two genomes on average (optimally selected, orange dashed line). **d** Percentage of genomes containing complete carbohydrate utilization pathways stratified by genera. Rows and columns are hierarchically clustered based on the presented data. Pathways that were not found in the analysis but have experimental evidence are shown in gray. Supplementary Data 5 provides the full list of genes analyzed for presence in each carbohydrate utilization pathway. The number of genomes per genus is as in Fig. 1b. **e** Schematic overview of L-arabinose utilization using low-affinity proton-motive force (PMF)-driven transporter or high-affinity ATP-binding cassette (ABC) transporters. **f** Low-affinity and high-affinity transporters for L-arabinose uptake seem to be mostly mutually exclusive in *Salmonella* and *Escherichia*, whereas both systems are present in *Citrobacter*.

A key component of colonization resistance is nutrient exploitation. This feature is influenced by the complexity of the microbiota and further enhanced by close relatives of the incoming strain, since these will feature a particularly high metabolic resource overlap^22,53^. As a proxy for understanding the metabolic resource profiles of *Enterobacteriaceae*, we investigated the presence of different carbohydrate utilization pathways. On average, *Enterobacteriaceae* genomes feature 31 different carbohydrate utilization pathways. The number of detected carbohydrate utilization pathways varied from 3 in a *Serratia symbiotica* strain to 40 in an *Escherichia coli* strain (Fig. 5a). We observed a weak correlation between genome size and the size of the metabolic resource profile for *Enterobacteriaceae* (Spearman’s correlation = 0.49, *P*-value ≤ 2.2E−16, Fig. 5b). This trend holds true for most analyzed genera and is particularly apparent in symbiotic *Enterobacteriaceae* species, which exhibit a limited metabolic resource profile correlating with their reduced genome size compared to non-symbiotic species (Spearman’s correlation = 0.89, adj. *P*-value ≤ 2.2E−16, Supplementary Fig. S2, Supplementary Data 7). To explore the link between microbiota (specifically *Enterobacteriaceae*) complexity and metabolic resource overlap, we assessed the number of strains minimally needed to achieve a high overlap in metabolic resources - defined as 90% of carbohydrate utilization pathways present. When selection was guided by carbohydrate utilization gene analysis (Figs. 2–4), a median of just 2 out of 19795 strains sufficed, typically including at least one *Klebsiella* or *Escherichia* strain. In contrast, selecting genomes based on the distribution of *Enterobacteriaceae* in healthy human guts required 7 strains, while selection normalized by the number of genomes per genus required a median of 15 strains (Fig. 5c).

To enhance visualization, we mapped the presence of carbohydrate utilization pathways across all 16 analyzed *Enterobacteriaceae* genera (Fig. 5d). Supplementary Data 6 details the complete list of enzymes included in each carbohydrate degradation pathway assessed. Transporters were generally excluded from this analysis since they often show low substrate specificity. Notably, *Klebsiella oxytoca* strains exhibit, on average, the highest number of carbohydrate utilization pathways, which likely contributes to their context-dependent ability to be competitive against *Salmonella* spp^22,55^. Additionally, our analysis can shed light on why some *Enterobacteriaceae* encode a low-affinity L-arabinose importer, while others possess a high-affinity version. *Salmonella* strains encode an α-N-arabinofuranosidase, which liberates L-arabinose from arabinan polymers and is important in long-term infection experiments^76^. L-arabinose can be transported by a low-affinity proton motive force (PMF)-driven transporter and a high-affinity ATP-binding cassette (ABC) transporter^60^ (Fig. 5e). Our analysis has revealed that in certain instances, members of the *Enterobacteriaceae* family have evolved to specialize in using one of these transport systems and have consequently lost the other. This observation correlates with the presence of α-N-arabinofuranosidase, indicating that *Enterobacteriaceae* that have acquired this hydrolase are able to locally increase L-arabinose concentrations to an extent that would render a high-affinity transport system dispensable (Fig. 5f).

In conclusion, our large-scale pan-genome analysis provides important insights into current questions in the microbiome field. A key goal is the active displacement of unwanted bacteria from the gut through the application of probiotic microbiota consortia^58^. Understanding metabolic resource overlap and how to rationally design these consortia to maximize this overlap is crucial for displacing problematic strains or using them as probiotics to enhance colonization resistance against such strains.

## Discussion

Due to the increasing availability of sequencing data, modern bioinformatic analysis has evolved from focusing on single model organisms to encompassing entire bacterial families. This shift allows for a comprehensive exploration of gene distribution, diversity, and genome plasticity. In our approach, we analyzed a large number of enterobacterial genomes to construct the pan-genome, identifying genes present in over 95% of genomes as core genes, while those found in fewer than 95% are classified as accessory genes^77,78^. The *Enterobacteriaceae* family, although not highly abundant in the mammalian gut, plays a disproportionately large role in public health due to some pathogenic members^1^.

This study sheds light on the organization and function of the core and accessory genomes within the *Enterobacteriaceae* family. Notably, we identified a substantial accessory genome comprising non-annotated or hypothetical gene families, even within well-studied organisms like *E. coli*. Remarkably, nearly 50% of *E. coli’s* genome remains either unannotated or only annotated with predicted functions, with around 30% of protein functions still experimentally unstudied^79,80^. For less-researched *Enterobacteriaceae* members, this proportion could be even higher. Recent findings in *E. coli* highlight that unknown gene families significantly complicate the pan-genome structure through co-occurrence or avoidance^81^. We expect similar influences within the *Enterobacteriaceae* pan-genome, where over 70% of gene families are hypothetical. In addition, specifically, the accessory genome can also be shaped by horizontal gene transfer (HGT)^25^. It has previously been shown that recently transferred genes are mostly part of the accessory genome. Their functions are often associated with resistance and defense mechanisms, intracellular trafficking and the mobilome, which can be indicative of the evolutionary pressures underlying HGT^82,83^. Furthermore, the absence of certain pathway components in our dataset may stem from incomplete, inconsistent or missing annotations, as gene annotation continues to present a key challenge in the analysis of prokaryotic genomes and pan-genomes^84,85^. Other potential issues in our study may arise from the chosen algorithms for gene family clustering, including the merging of some gene families due to high sequence similarity, unresolved gene fusions, or incomplete fission events. Collectively, these challenges can obscure a comprehensive understanding of pan-genome dynamics.

While our study primarily focuses on the pan-genome of *Enterobacteriaceae* at the family level, the extensive dataset also allows us to explore pan-genome structure at a finer taxonomic resolution. We constructed gene families at various amino acid sequence identity thresholds to accommodate variation in evolutionary rates among gene families^86^. For more detailed pan-genome analysis within specific *Enterobacteriaceae* species, stricter sequence identity thresholds may be required to account for the narrower taxonomic range. To define species-level pan-genomes, fixed thresholds are commonly used in the literature^87^. Our dataset’s taxonomic classification relies on NCBI annotations, which we validated using 120 marker genes with GTDB-Tk. While genus-level classifications are generally consistent, we observed some discrepancies at the species level, particularly within the genus *Yersinia*, which may affect higher-level analyses. The current pan-genome analysis predominantly focuses on well-studied genera such as *Escherichia*, *Klebsiella/Raoultella*, and *Salmonella*, reflecting current sequencing biases. For example, over 95% of available *Enterobacteriaceae* genomes in ProGenomes3 originate from these genera^38^. As metagenomics and the availability of metagenome-assembled genomes (MAGs) continue to advance, we anticipate broadening this focus. This progress will enhance our understanding of gene presence and absence in a population context. Ideally, comparing genomes from various niches will allow us to define enterobacterial ecotypes - strains that use a similar ecological niche - and identify niche-specific genes^88^, providing insights into evolutionary advantages in specific ecological or genetic contexts.

Our study utilized energy metabolism to exemplify how a pan-genome analysis at the family level of *Enterobacteriaceae* enhances our understanding of a key feature of the microbiome: nutrient exploitation. As facultative anaerobes, metabolic genes that enable *Enterobacteriaceae* to thrive in both aerobic and anaerobic environments are part of the core genome. This includes glycolysis, Entner-Doudoroff and the (non)-oxidative pentose phosphate pathway, as well as tricarboxylic acid (TCA) cycle and mixed acid fermentation (Figs. 3 and 4c). Noteworthy, most of the pathways required to utilize specific carbohydrates and most terminal reductases are found in the accessory genome, likely reflecting their high context-dependence, i.e., different environmental availability of the respective substrates in different niches. Recent publications have highlighted the availability of carbohydrates in the mammalian intestine, showing that carbohydrates whose degradation belongs to the core genome are also abundant in the gut^89,90^. This observation largely reflects the sugar monomeric composition of diet, with D-glucose, D-fructose, D-galactose, and D-mannose being the most abundant^91–93^. Inorganic electron acceptors play a crucial role, particularly during enteric diseases or microbiota disturbances. However, the availability of these molecules is largely context-dependent. In the gut, inflammation-induced oxidative stress is necessary to generate nitrate and tetrathionate, which favor the growth of *Enterobacteriaceae*^94,95^. At the same time, oxygen becomes available due to the host’s metabolic shift^96–98^. In contrast, fumarate respiration, a core metabolic pathway, is always used and contributes to *Salmonella* growth in the non-inflamed and inflamed gut because fumarate can be supplied through multiple sources^99–103^. This is essential because biosynthetic pathways, such as heme *b* and pyrimidine synthesis, rely on fumarate reductase and quinones as electron acceptors under anaerobic conditions. The importance of fumarate respiration is particularly evident in the genera *Escherichia* and *Salmonella* during all stages of infection^89,90,101–104^.

Freter’s nutrient niche theory posits that a bacterial species needs to more efficiently utilize a nutrient niche than the competition^15,16^. This theory can translate into differences in bacterial metabolic capacity, such as the presence of genes that allow one species to use a carbohydrate inaccessible to others. Our analysis highlights the high variability in carbohydrate utilization systems among *Enterobacteriaceae* and provides a framework for understanding why different *Enterobacteriaceae* strains can often co-exist in the same intestine^105^. The sheer abundance of pathways per strain may also explain why *Klebsiella* strains are repeatedly discovered as excellent probiotics for limiting *Salmonella* colonization in certain contexts^22,55^. Our pan-genome analysis provides important groundwork for the future design of probiotic consortia aimed at efficient nutrient blocking or exploitation. By maximizing metabolic resource overlap, these consortia could effectively inhibit problematic bacteria that infiltrate a healthy, homeostatic gut microbiome. Interestingly, on average, the highest number of carbohydrate utilization pathways was found in an *Escherichia coli* strain, with 40 pathways. It is important to note that defining metabolic capabilities based on bioinformatic analysis has limitations, as the presence of a gene does not necessarily imply the production of a functional protein, as previously demonstrated in *Salmonella enterica* serovars Typhi and Paratyphi A^106^. No strain possessed all 49 analyzed carbohydrate degradation pathways, raising questions about the cost-benefit balance that governs the number of carbohydrate utilization pathways within each *Enterobacteriaceae* species and why *Klebsiella* harbors so many.

In conclusion, Freter’s nutrient niche theory can be differentiated into shared resources, accessible to various community members, and niche-defining nutrient sources, available to only one particular strain. The former nutrient sources, as evidenced by our pan-genome analysis, will likely include D-glucose, D-glucose epimers, D-glucose-containing disaccharides, and modified D-glucose molecules. In principle, these are nutrient sources that every *Enterobacteriaceae* strain can utilize. Niche-defining nutrient sources are exclusive, accessible by only one bacterial species but not by its competitors; this can provide the decisive advantage for successful colonization. Our carbohydrate-focused genome analysis strategy may offer a rational approach to select strains for decolonization therapies or mitigating infection risks.

## Methods

### Selection of *Enterobacteriaceae* genomes

From the Progenomes v3 database^38^, 525,581 genomes classified as *Enterobacteriaceae* or *Yersinia* according to the NCBI taxonomy were downloaded. First, genomes were screened to select high-quality genomes. In a first step, CheckM2^107^ assembly statistics, kindly provided by Sebastian Schmidt, were used to remove genomes with completeness below 95% and contamination larger than 10%. Furthermore, highly fragmented genomes (more than 300 contigs) were discarded. Next, genomes with total contig sizes larger or smaller than 1.5 * IQR (interquartile range) compared to genomes of the same species were removed. Additionally, genomes with a coding density outside of the 0.5–1.5 range were not considered. Finally, all genomes of species containing fewer than 10 genomes in the filtered dataset were removed, and overrepresented species (mainly *Escherichia coli*, *Salmonella enterica* and *Klebsiella pneumoniae*) were subset uniformly at random without considering maintenance of original diversity to maximally 3200 genomes to generate an *Enterobacteriaceae* genome collection consisting of a total of 19,795 genomes. All genera are represented by at least 7 different BioProjects and have a median representation of 73 BioProjects (Supplementary Fig. S3). Unless otherwise stated, the NCBI taxonomic classification was used to assign taxonomy to each genome. The identifiers and taxonomic classification of all genomes are provided in Supplementary Data 1.

### Pan-genome analysis

All genomes were preprocessed with Prodigal v2.6.3^108^ to obtain coding sequences (CDS). We generated a family-wide pan-genome by clustering CDS into gene families using PIRATE v1.0.5 using standard parameters and an MCL inflation parameter of 3. Overall, 141,675 gene families were created. Ubiquity was calculated for each gene family and for each genus based on presence and absence. In short, for each genus, the number of genomes containing a specific gene family was divided by the total number of genomes for the specific gene family. Taxonomic classification was based on the provided NCBI taxonomy. Gene families were labeled as core if they occurred in at least 95% of all investigated genomes (core genome). All remaining gene families were classified as the accessory genome. Accessory gene families, which were present only in genomes of the same genus, were labeled as genus-specific, while gene families present in at least 2 genera were labeled as genus-unspecific. The core genomes comprised 1801 gene families (1.3%) while the accessory genome consisted of 1,39,874 (98.7%) gene families.

We annotated all gene families against the UniProtKB/Swiss-Prot database^40^ using MMseqs2 v14.7e284 (--start-sens 1 --sens-steps 3 -s 7 –max-accept 10000). Hits with e-value > 1e − 5 were discarded, and the best hit was kept. Annotations were aggregated per gene family, and if available, the two most abundant annotations were reported. Sequences that were not annotated were labeled as ‘unknown’. Based on the annotation, gene families were summarized into three annotation status brackets based on the inferred short gene name: Resolved (≥90% of the sequences contain the same annotation), Ambiguous (<90% of the sequences contain the same annotation and less than 10% Unknown) and not annotated (≥10% of the sequences are not annotated). For annotated gene families, we selected a representative sequence uniformly at random from those with the most common annotation. For non-annotated gene families, the representative sequence was chosen uniformly at random from all sequences in the gene family.

A phylogenetic tree was constructed based on core gene families present in at least 99% of all genomes. Additionally, families with unresolved annotations or families containing non-single copy were removed. The full list is provided in Supplementary Data 3. The resulting 103 gene families were subsequently used in the phylogenetic analysis. Briefly, each gene family was individually aligned using Muscle v5.1^109^ and SNP-sites v2.5.1^110^ was used to produce alignments of variant sites. The resulting alignments were concatenated, and a maximum-likelihood tree was constructed using RAxML-NG^111^ with the general time-reversible model GTR + G model with a discrete GAMMA model of rate heterogeneity with 4 categories. ITOL v6.8.1^112^ was used to midpoint root the tree and for tree visualizations. Peripheral members of *Enterobacteriaceae* are shaded orange and include *Yersinia*, *Serratia*, and *Plesiomonas*^1^.

### Mapping the core and accessory genomes on metabolic pathways

The *E. coli* K-12 database EcoCyc.org was utilized as a primary point of reference for metabolic pathways^113^, and EcoSal Reviews^75,114,115^. The pathways presented in these figures (Figs. 2–4) are a curated selection of annotated and referenced research, primarily grounded on *E. coli* and *Salmonella* spp. research. A full list of abbreviations is available in Supplementary Data 4. Complexes, especially in Fig. 4, are named by their operon rather than individual genes. A complete list of all genes within each complex is provided in Supplementary Data 5.

### Investigating the distribution of carbohydrate utilization pathways

We searched the pan-genome for gene families containing annotations presented in Fig. 3. A complete list can be found in Supplementary Data 6. We consider a carbohydrate utilization pathway to be present only if each of its genes can be found within a specific genome. Due to the potential substrate ambiguity of transporters and to avoid making pathway-specific assumptions about substrate specificity, we limited our analysis to intracellular metabolic genes. The only exception was glucose utilization, where the transporter PtsG is the main entry point into glycolysis. Spearman correlation values of genome size against the number of carbohydrate utilization pathways present were computed using the cor.test function of stats v4.1.3 in R. The core and accessory status of a carbohydrate utilization pathway is assessed based on the average presence of each gene in all genomes. A pathway is considered ‘core’ if the average is larger than or equal to 95%, and considered ‘accessory’ otherwise. Genera and carbohydrate utilization pathways are ordered based on a hierarchical clustering performed using Euclidean distance and complete linkage as implemented in the ComplexHeatmap package in R^116^.

To calculate the metabolic resource overlap of subsampled genomes, we considered the presence of any alternative pathway for a given sugar molecule as sufficient to mark that resource as covered - with the exception of L-ascorbate, fructoselysine, glycerol, sucrose, and trehalose, for which we differentiated between specific pathways (e.g., L-ascorbate (aerobic) and L-ascorbate (anaerobic)), as indicated on the *x*-axis (Fig. 5d). This approach resulted in a total of 49 defined metabolic resources (Supplementary Data 6). We distinguish between three different subsampling methods: “optimally selected”, “random” and “human adult gut-like” and calculate metabolic resource overlap individually for each method. For the “optimally selected” subsampling, we randomly selected an initial genome as a starting point. Subsequently, the next genomes are chosen to cover most of the missing resources until at least 90% are covered. In random subsampling, a random genome was chosen as a starting point. Then, we randomly selected a genome without replacement at each step until at least 90% of the metabolic resources are covered. To account for the uneven distribution of genera in the dataset, each genus was assigned an equal selection probability. To subsample according to a human adult gut distribution of *Enterobacteriaceae*, we obtained healthy human gut metagenomic samples from the Unified Human Gastrointestinal Genome (UHGG) v.1.0 catalog^117^. To this end, we extracted healthy adult human samples (*n *= 5128) and read counts of non-*Enterobacteriaceae* species were removed. Additionally, genera not represented in our data set were excluded. Based on this filtered data set, read counts were aggregated by genus, and average abundances were calculated by sample. Subsampling was implemented by first selecting a genus based on previously calculated average abundances, followed by randomly selecting a genome from the chosen genus. This process was repeated until at least 90% of the metabolic resources were covered.

To visualize the presence of the high- and low-affinity L-arabinose transport systems and their correlation with the presence of an α-N-arabinofuranosidase, we randomly selected in total 100 *Citrobacter*, *Escherichia* and *Salmonella* genomes. Based on the retrieved annotation, we selected gene families annotated with *araF*, *araG* and *araH* for the high-affinity ABC transport system, *araE* and *araF43*a for the low-affinity PMF transporter, and *araA*, *araB* and *araD* for the intracellular pathway. Rare gene families and singletons containing any of the aforementioned annotations are not shown. Genomes and gene families are ordered based on a hierarchical clustering as provided in the ComplexHeatmap package in R using Euclidean distance and complete linkage^116^.

### Reporting summary

Further information on research design is available in the Nature Portfolio Reporting Summary linked to this article.

## Supplementary information

Supplementary Information

Description of Additional Supplementary Files

Supplementary Data 1-7

Reporting Summary

## Data Availability

A summary of each gene family created with PIRATE, along with all analyzed *Enterobacteriaceae* genomes and their associated gene family memberships in GFF3 format, is deposited in Zenodo at 10.5281/zenodo.14849847 (ref. ^118^).

## Code availability

The raw data and R code used to generate the figures in this study are available at 10.5281/zenodo.14849847 (ref. ^118^). The parameters used for the analyses and the software versions are detailed in the “Methods” section.
