# Supplementary material for: Gene-level analysis of core carbohydrate metabolism across the Enterobacteriaceae pan-genome
Source: Commun Biol. 2025 Aug 18;8:1241. doi: 10.1038/s42003-025-08640-5 (PMC12361417; doi:10.1038/s42003-025-08640-5)

Title:

Gene-level analysis of core carbohydrate metabolism across the *Enterobacteriaceae* pan-genome

Nicolas Näpflin[1*], Christopher Schubert[2*], Lukas Malfertheiner[1], Wolf-Dietrich Hardt[2], and Christian von Mering[1]

Affiliations:

[1]Department of Molecular Life Sciences and Swiss Institute of Bioinformatics, University of Zurich, Zurich, Switzerland

[2]Institute of Microbiology, D-BIOL, ETH Zurich, Zurich, Switzerland

*These authors contributed equally

Correspondence: hardt@micro.biol.ethz.ch (W.-D.H), mering@mls.uzh.ch (C.v.M)

Supplementary Information

Figure S1. **Phylogenetic reconstruction of the *Enterobacteriaceae* family based on 103 concatenated core genes (present in at least 99% of investigated genomes).** The tree is midpoint rooted, and the outer ring represented taxonomic classification based on the NCBI taxonomy. Genomes not classified to genus level are labelled as ‘Other’. Peripheral members of *Enterobacteriaceae* (*Yersinia, Serratia and Plesiomonas)* are shaded orange. The color legend contains the number of different species per genera in brackets. Genome counts per species are recorded in **Supplementary Data 2.** The concatenated core genes used are listed in **Supplementary Data 3.** The scale bar represents estimated nucleotide substitutions per site.

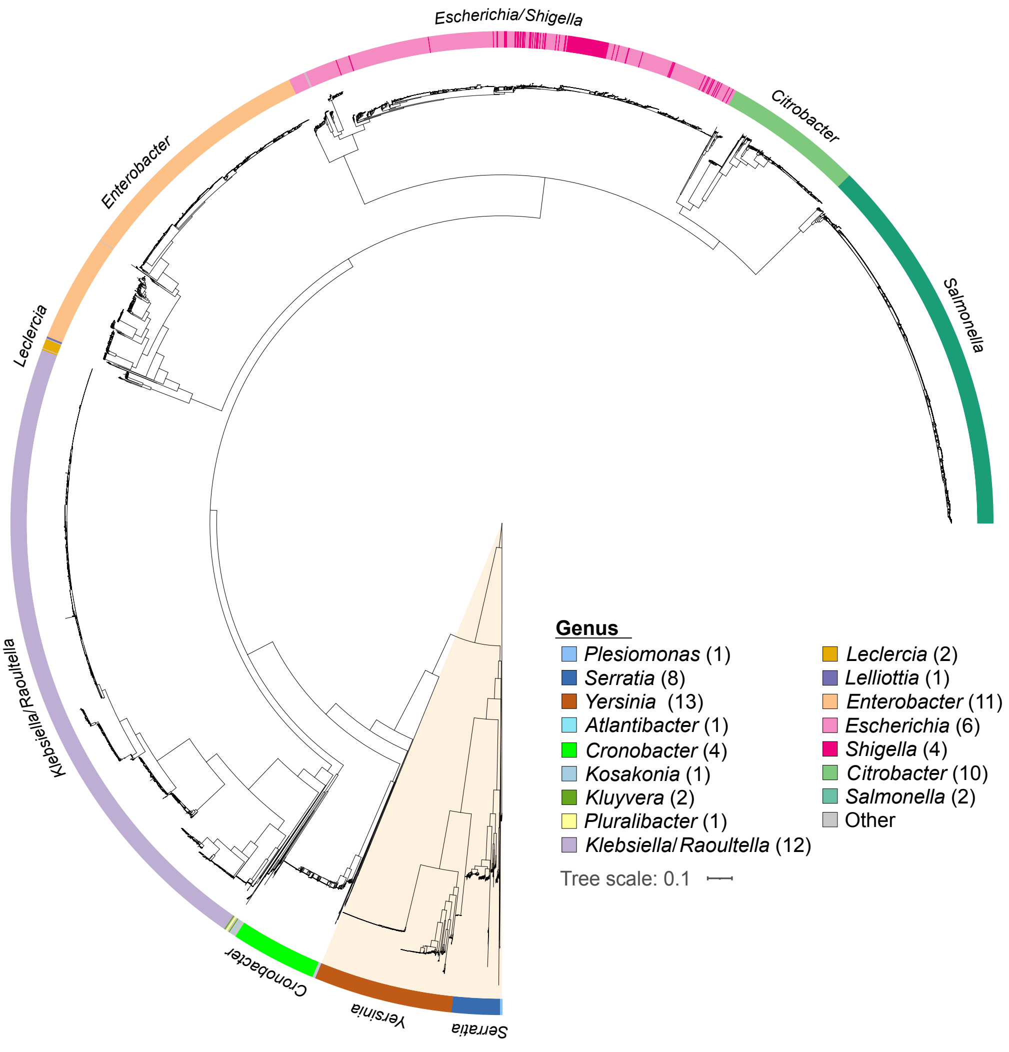

Figure S2. Symbiotic *Enterobacteriaceae* species exhibit a reduced metabolic resource profile. Symbiotic *Serratia* species exhibit a reduced metabolic resource profile, measured as number of carbohydrate utilization gene present, as well as a reduced genome size compared to non-symbiotic *Serratia* species.

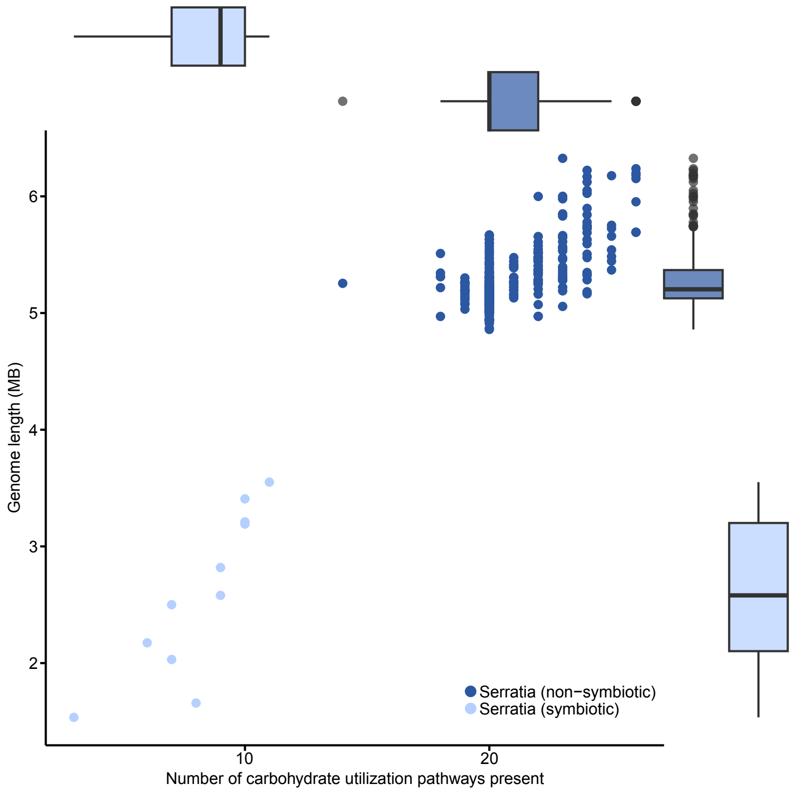

Figure S3. Distribution of BioProjects. a Contribution of the three largest and all remaining ones to *Enterobacteriaceae* genomes in ProGenomes3 and to the selected subset used in this study. **b** The number of different BioProjects is shown for each genus. The three most represented genera are labeled. Data are shown as median (black vertical line) and 25% and 75% percentiles (hinges). Whiskers extend from the hinges to the maxima and minima, no further than 1.5× distance of the interquartile range.

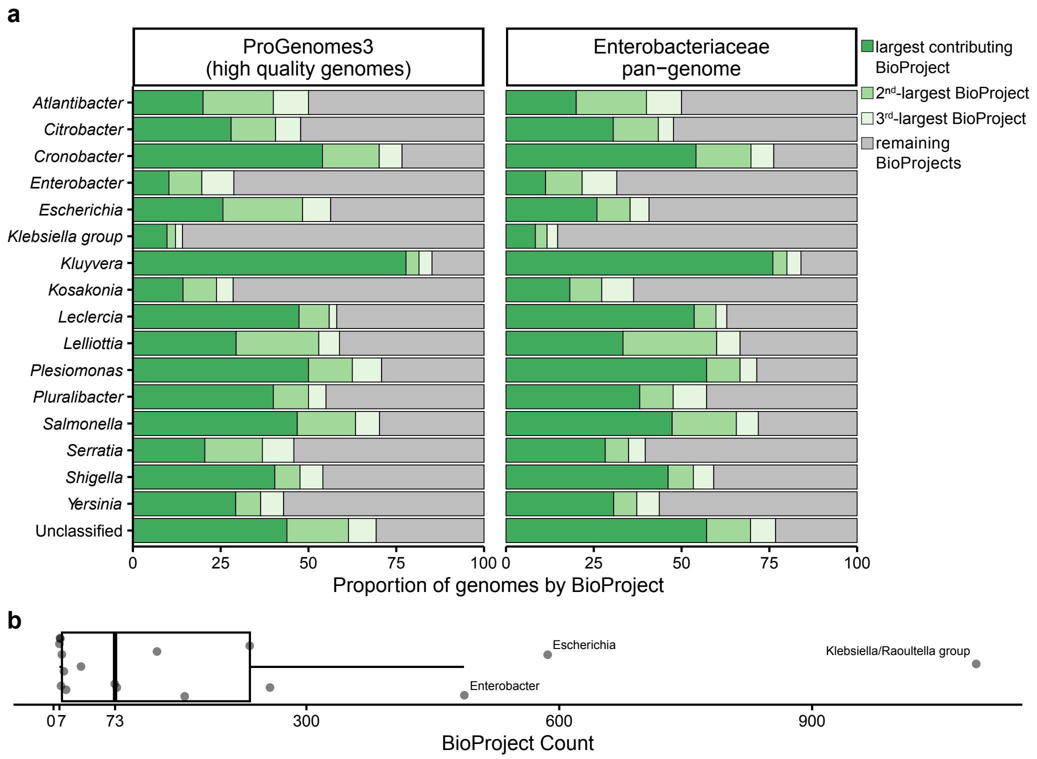

Supplement: Supplementary file 1 — Supplementary Information [file 42003_2025_8640_MOESM1_ESM.pdf]
