# Supplementary material for: Gene-level analysis of core carbohydrate metabolism across the Enterobacteriaceae pan-genome
Source: Commun Biol. 2025 Aug 18;8:1241. doi: 10.1038/s42003-025-08640-5 (PMC12361417; doi:10.1038/s42003-025-08640-5)
Supplement: Supplementary file 2 — Description of Additional Supplementary Files [file 42003_2025_8640_MOESM2_ESM.docx]

Description of Additional Supplementary Files

**File name:** Supplementary Data 1

**Description:** Genome accessions for *Enterobacteriaceae* genomes used.

**File name:** Supplementary Data 2

**Description:** Genome counts per species included in the study.

**File name:** Supplementary Data 3

**Description:** Gene families used for core tree.

**File name:** Supplementary Data 4

**Description:** Abbreviations.

**File name:** Supplementary Data 5

**Description:** Full gene list of complexes.

**File name:** Supplementary Data 6

**Description:** Full gene list of carbohydrate utilization genes.

**File name:** Supplementary Data 7

**Description:** Correlation of genome size versus number of carbohydrate utilization pathways.
